# An Outcome Analysis of Pediatric Diaphyseal Fractures Treated Surgically With the Titanium Elastic Nailing System

**DOI:** 10.7759/cureus.59716

**Published:** 2024-05-06

**Authors:** Nikhil Warade, Supratim Roy, Aliasgar Moaiyadi, Bhavesh Patidar, Chandrashekhar M Badole

**Affiliations:** 1 Department of Orthopaedics, Mahatma Gandhi Institute of Medical Sciences, Wardha, IND

**Keywords:** pediatric, nail, long bone, flynns criteria, tens, diaphyseal, fractures

## Abstract

Introduction

Pediatric fractures account for one-fourth of all pediatric injuries. Stabilizing the fracture, regulating the length and alignment, encouraging bone healing, and minimizing morbidity and problems for the child and family are the objectives of treatment for diaphyseal fractures of long bones in children. Our goal is to investigate how pediatric diaphyseal long bone fractures are treated with a titanium elastic nailing system (TENS).

Methods

A prospective interventional study was conducted on 24 children who had displaced diaphyseal fractures of major long bones, involving 31 diaphyseal fractures of long bones. Utilizing Flynn's grading standards, the result was examined.

Results

The mean age was 12.20 years. The youngest child was seven years old and the eldest child was 16 years old. There were 20 boys (83.33%) and four girls (16.67%). The male-to-female ratio was noted to be 5:1. The commonest mode of injury was road traffic accidents (12 cases, 50%), followed by falls while playing (10 cases, 41.67%). Other causes included falls from height (one case, 4.17%) and blunt trauma (one case, 4.17%). The commonest bone to get fractured was the femur (37.50%), followed by both bones of the forearm (29.17%), tibia (20.83%), humerus (8.33%), and ulna alone (4.17%). The middle third (21 fractures, 67.74%) was the most prevalent location for fractures. Five fractures each (16.13%) accounted for in the proximal and distal thirds. Twelve fractures (38.71%) were detected on the left side, while the majority of fractures (19 fractures, 61.29%) were seen on the right side. Most of the fractures in this group were transverse fractures (18 fractures, 58.06%) followed by oblique fractures (eight fractures, 25.81%). Comminuted fractures accounted for five fractures (16.13%). Of the 31 fractures, open reduction had to be done in two fractures, after unsuccessful attempts at closed reduction. Closed reduction was done in 29 fractures. There were 15.12 weeks in the average union term. The range is six weeks to 39 weeks. The most frequent side effect was discovered to be skin irritation at the entry site. The extraosseous portion of nails caused irritation at two entry sites (6.45%). A case had delayed union (3.23%) and restricted knee range of movements.

Conclusion

For the treatment of juvenile diaphyseal fractures of the long bones, the TENS is the best option. It is a quick, straightforward, safe, dependable, and efficient way to treat pediatric long-bone fractures in patients aged five to 16 years. The healing process takes a fair amount of time, while the surgery takes less time. It does away with the necessity for extended bed rest and significantly shortens hospital stays. It provides stability and elastic mobility, which is perfect for early mobilization and quick union at the fracture site. It has a low rate of complications and produces excellent functional results.

## Introduction

Injuries among children have become a major global public health issue. Several thousand youngsters are thought to sustain non-fatal injuries and experience differing degrees of disability [1]. Fractures account for 10-25% of pediatric injuries, according to estimates [2]. The rise in traffic accidents and sports engagement is the primary cause of the rising number of fractures among youngsters [3]. The child's age, the severity and pattern of the fracture, and the surgeon's or the institution's preferences can all have a significant impact on the treatment plan; fixing juvenile diaphyseal fractures should ideally result in an "internal splint" that distributes weight, keeps the fracture smaller until a hard callus forms, and does not jeopardize with the growth regions [4]. It is appropriate to use surgical therapies to prevent extended immobilization, as well as physical, social, and psychological consequences. These consist of elastic stable intramedullary nailing, fixation with plates and screws, external fixators, and intramedullary nailing. Submuscular plating is an extensive procedure. Intramedullary nailing can damage the physis. In order to prevent injury to the physis, elastic stable intramedullary nailing has gained popularity as a treatment for pediatric diaphyseal shaft fractures [5]. For the treatment of femoral shaft (long bone) fractures, the titanium elastic nailing system (TENS) appears to be a more physiologically sound and successful approach [6]. The technique is straightforward, quick, and safe, and it has the benefits of early union, early mobilization, and an early return to function with little problems [7]. Our aim is to study the outcome of diaphyseal pediatric long-bone fractures managed with TENS.

## Materials and methods

This was a prospective interventional study done on a total of 31 diaphyseal fractures of long bones in 24 children who were treated surgically in the Department of Orthopedics between July 2020 and August 2022. Children between the ages of five and 16 with open growth plates coming to the Orthopedics outpatient department/casualty with long-bone fractures and managed surgically with TENS were included in this study, either through closed or open reduction.

Inclusion and exclusion criteria

The various inclusion criteria include children from five years to 16 years of age with open physis of long bones, children of both sexes, with closed diaphyseal fractures, patients fit for surgery, and willing for surgery. The exclusion criteria include patients who have polytrauma and compound fractures.

Methodology

This study has used titanium elastic nails (6% aluminum and 7% niobium alloy) in all patients. All operated patients were called for follow-up at three weeks, six weeks, 12 weeks, 24 weeks, and nine months. All these findings were recorded in the proforma.

Statistical analysis

Chi-square tests of significance were used in the statistical study, which included both descriptive and inferential statistics. Graph Pad Prism 7.0 (GraphPad Software, San Diego, USA) and IBM SPSS Statistics for Windows, Version 27 (Released 2020; IBM Corp., Armonk, New York, United States) were the software versions utilized for the analysis, and a level of significance of p < 0.05 was applied.

The final outcome was assessed by Flynn's scoring criteria as described below in Table 1 [8]. The parameters included in this criterion are limb length inequality, malalignment, pain, and complications. 

**Table 1:** Flynn’s scoring criteria [8]

Parameter/result	Excellent	Satisfactory	Poor
Limb length inequality	<1.0 cm	<2.0 cm	>2.0 cm
Malalignment	50	100	>100
Pain	None	None	Present
Complications	None	Minor and resolved	Major/lasting morbidity

## Results

The observations of this study are based on 31 diaphyseal fractures in long bones among 24 children who were treated surgically at the Department of Orthopedics between July 2020 and August 2022.

**Figure 1:**
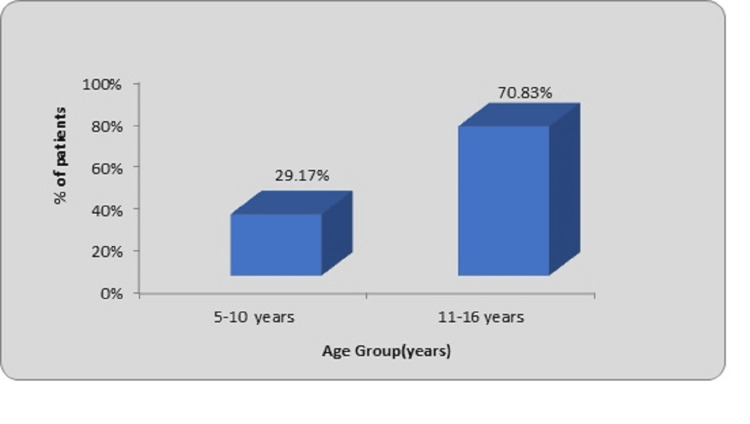
Age-wise distribution of patients

The majority of the patients (17, 70.83%) belonged to the 11-16 age range. There were seven kids between five and 10 years old. The average age was found to be 12.20 years as shown in Figure 1.

**Figure 2:**
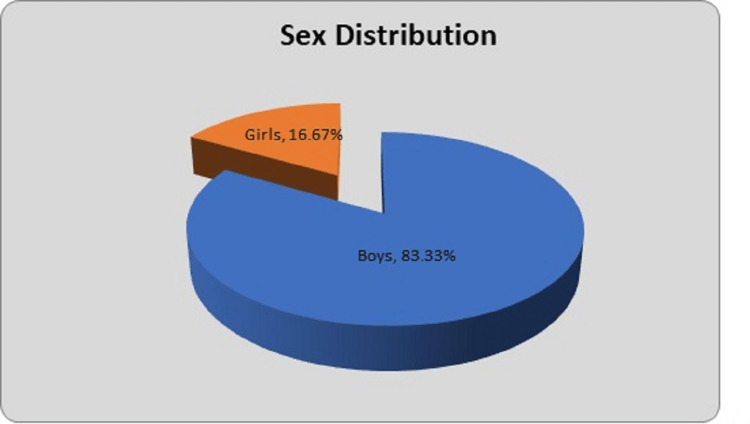
Gender-wise distribution of patients

There were 20 boys (83.33%) and four girls (16.67%) as shown in Figure 2. The male-to-female ratio was noted to be 5:1.

**Figure 3:**
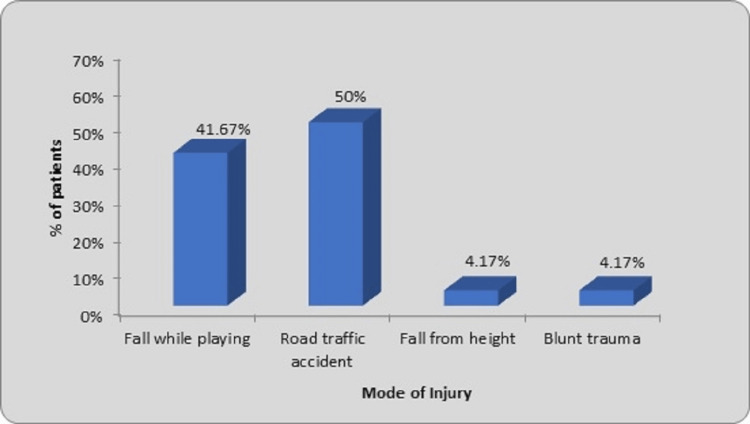
Distribution of patients according to mode of injury

A higher number of physical activities may be responsible for the higher number of male children. The commonest mode of injury was road traffic accidents (12 cases, 50%), followed by falls while playing (10 cases, 41.67%). Other causes included falls from height (one case, 4.17%) and blunt trauma (one case, 4.17%) as shown in Figure 3.

**Figure 4:**
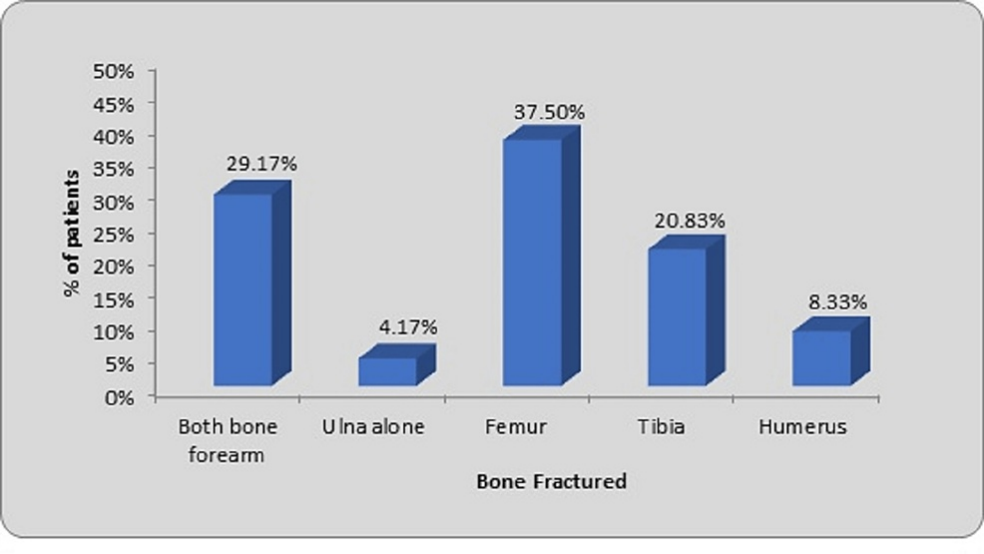
Distribution of patients according to bones fractured

The commonest bone to get fractured was the femur (37.50%), followed by both bones of the forearm (29.17%), tibia (20.83%), humerus (8.33%), and ulna alone (4.17%) as shown in Figure 4.

**Figure 5:**
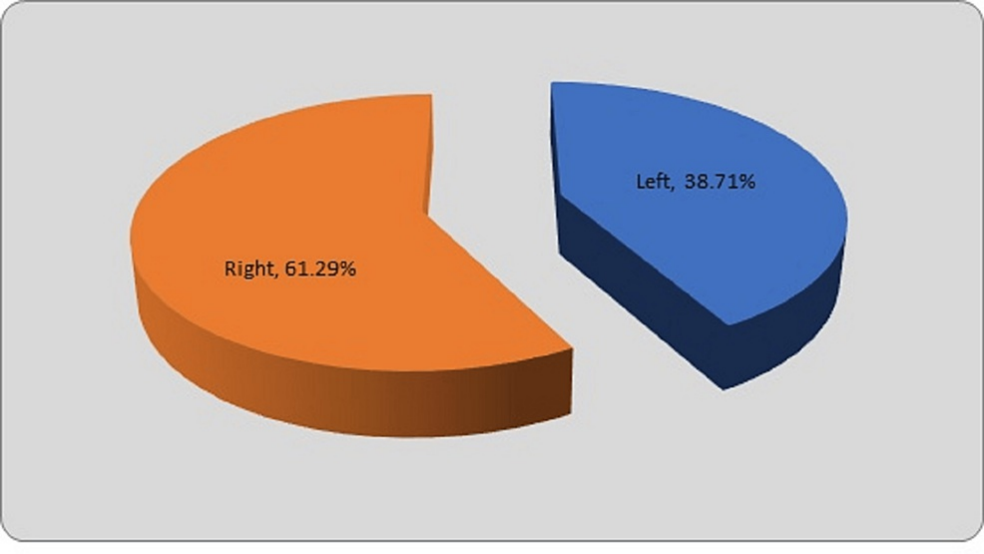
Distribution of fractures according to the side of injury

The middle third (21 fractures, 67.74%) was the most prevalent location for fractures. Five fractures each (16.13%) accounted for in the proximal and distal thirds. Twelve fractures (38.71%) were detected on the left side, while the majority of the fractures (19 fractures, 61.29%) were seen on the right side as depicted in Figure 5.

**Figure 6:**
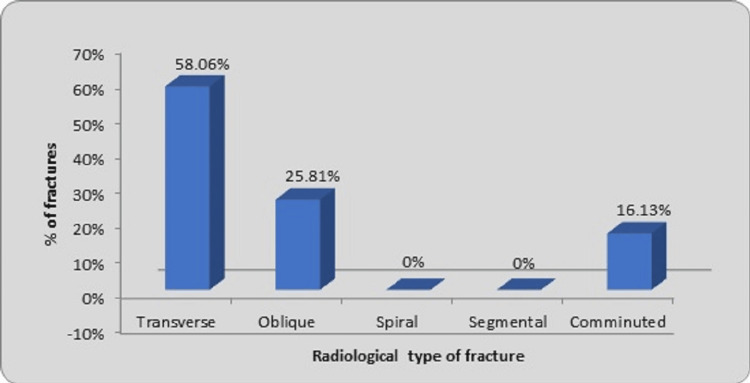
Distribution of fractures according to radiological type of fracture

Transverse fractures accounted for a major proportion of this series (18 fractures, 58.06%) followed by oblique fractures (eight fractures, 25.81%) while comminuted fractures accounted for five fractures (16.13%), as observed in Figure 6.

**Figure 7:**
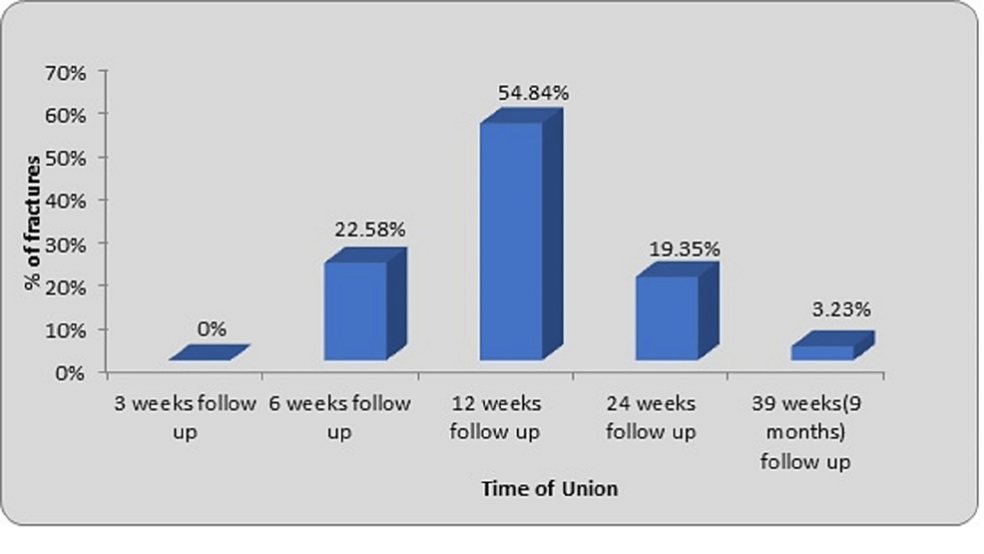
Distribution of fractures according to period of union for fractures

A total of 18 cases (75%) came to the hospital on the same day of trauma. Five cases (20.83%) arrived at the hospital between one and 10 days after the trauma, while one case (4.17%) arrived 10 days after the trauma. The mean interval between the trauma and presentation to the hospital was found to be 1.08 days. The mean interval between the admission and surgery was 5.33 days. The mean interval between the trauma and surgery noted was 6.41 days. Of the 31 fractures, open reduction had to be done in two fractures after unsuccessful attempts at closed reduction. Closed reduction was done in the remaining 29 fractures. The mean period of union was 15.12 weeks. The range is between six and 39 weeks as shown in Figure 7.

**Figure 8:**
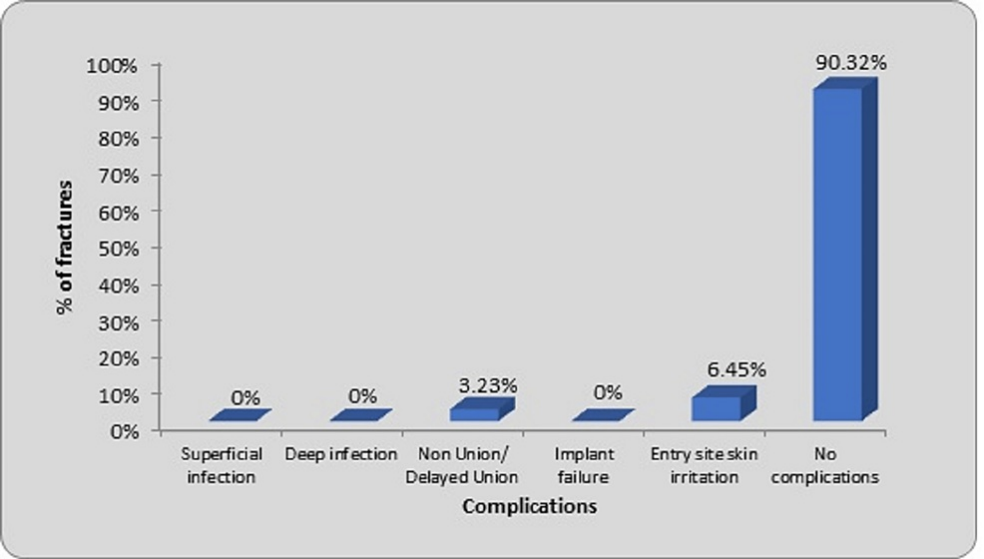
Distribution of fractures according to complications

The distribution of fractures according to various complications can be seen in Figure 8. The extraosseous portion of nails caused irritation at two entry sites (6.45%), which was relieved after analgesics. This was a minor complication that resolved completely. These two cases also had a restricted range of movements in the knee joint which improved with physiotherapy. One case had delayed union (3.23%) and restricted knee range of movements. Except for this case, all other cases had a full range of adjacent joint movements at the union. We did not encounter any case of deep infection or implant failure in our study.

**Figure 9:**
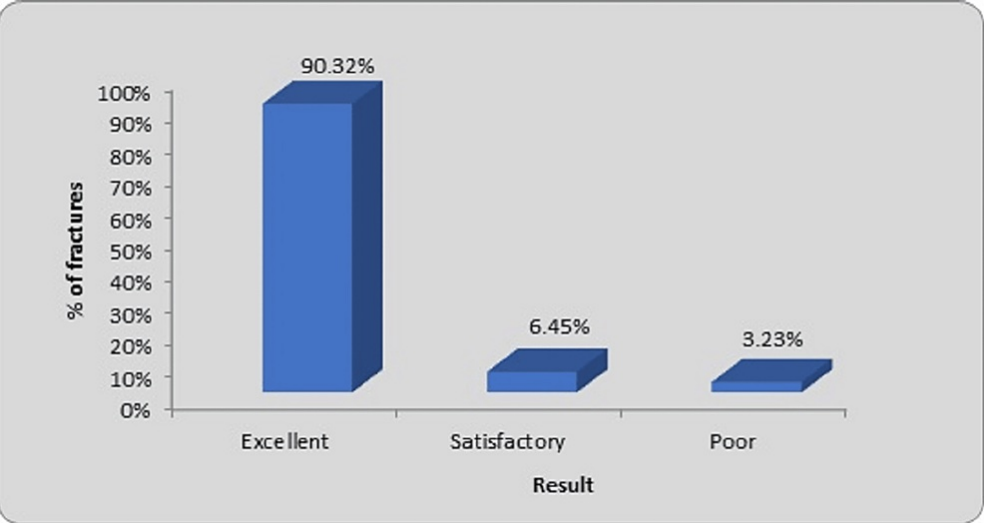
Evaluation of results

We have assessed the findings of our study which can be seen in Figure 9 using the scoring standards outlined by Flynn et al. [8]. It was found that 28 fractures, or accounting for 90.32% of all fractures, had favorable (excellent) outcomes for the most part. Two fractures (6.55%) healed satisfactorily, while only one fracture (3.23%) had a bad (poor) result.

## Discussion

The most common surgical technique for treating pediatric long bone fractures is not crossing the epiphyseal growth plate, which prevents growth disruptions and carries a low risk of sequelae [8]. In the current investigation, the predominant demographic comprised 17 patients within the age range of 11-16 years, constituting 70.83% of the study population. Additionally, seven patients (29.17%) fell within the age group of five to 10 years. The youngest participant was seven years old, while the oldest individual was 16 years old. The average age across the sample was calculated to be 12.20 years. This distribution of age groups reflects the diversity within the study cohort, providing a comprehensive overview of the population under consideration. Similar results related to the mean age were observed by Sankar et al. and Furlan et al. [9,10]. Sankar et al. observed the average age of the patient as 12.2 years (7.2-16 years range) [9]. Furlan et al. observed the average age of the patient as 11.7 years [10]. Baig et al. observed the age group 13 to 16 years old ranked second (33.3%) [11]. Raut et al. reported a distribution of patients in their study, revealing that the highest percentage, 36.7%, fell within the age range of six to eight years [12]. Following closely, 33.3% of patients were in the age group of nine to 11 years, while 30% were within the 12-14 years age bracket. This breakdown underscores the diverse age representation within the participant cohort and provides valuable insights into the demographics of the population under examination. In the study by Gavaskar and Singh, 16 cases (53%) were observed in the age group of nine to 12 years, while 14 cases (47%) were observed in the five to eight-year age group [13]. Naorem and Temjensunep noticed that this is mainly because physical activity in children increases with age, thereby increasing the risk of sustaining a fracture [14]. Flynn et al. had a mean age of 9.5 years (range four to 16 years), while Rennie et al. noticed an overall average age was 9.7 years [8,15]. Sarkar et al. observed an average age of eight years (range of six to 14 years) [16]. Bandyopadhyay noticed a mean age of 7.5 years (four to 12 years range), while Khuntia et al. observed a mean age of 9.3 years (range of six to 15 years) [17,18].

In our study, there were 20 boys (83.33%) and four girls (16.67%). The male-to-female ratio was noted to be 5:1. Many studies observed male preponderance. Raut et al. observed male preponderance (63%) [12]. Verma et al. observed that the boy-to-girl ratio was 2.35:1 (151 were boys and 64 were girls) [19]. In the study of Fernandez et al., there were 354 boys (64%) and 199 girls (36%) [20]. The study by Sarkar S et al. observed that 53 (74.28%) were boys and 18 (25.72%) were girls [16]. The male-to-female ratio was 2.89:1. In their study, Naorem and Temjensunep found that there were 22 males (73.33%) and eight females (26.67%) [14]. Ghilley et al. observed 64% boys and 36% girls [21]. In his study, Choudhury found 25 males and 11 females with a male-to-female ratio (2.3:1) [22]. Kumar et al. noticed 14 (70 %) males and six (30%) females, while Kayaokay and Aktuglu noted an equal distribution, with 15 females (50%) and 15 males (50%) [23,24].

In our study, road traffic accidents were the most common mode of injury (12 cases, 50%) followed by falls while playing (10 cases, 41.67%). Fall from height accounted for one case (4.17%), while blunt trauma also accounted for one case (4.17%). Several studies have noted road traffic accidents as the most common cause of injury. Gavaskar and Singh observed road traffic accidents were the mode of injury in 12 patients (40%) while falling from height in 18 patients (60%) [13]. According to Raut et al., 20% of fractures were caused by falls, while 80% of fractures were caused by motor accidents [12]. Ghilley et al. noted that falls from height occurred in four instances (28%), while automobile accidents accounted for eight cases (60%) of fractures [21]. Choudhury reported that the majority of fractures (69.44%) were attributed to traffic accidents, while the remaining fractures were caused by falls from height and injuries sustained during play [22]. Others, however, noticed harm modes other than the prevalent one of motor accidents. In their investigation, Naorem and Temjensunep observed that 21 instances (or 70%) were ascribed to unintentional falls, five (16.67%) to traffic accidents, and four (13.33%) to falls from height [14]. In their study, Reddy and Dhaniwala found that among the 28 cases, the most common mode of injury was a fall while playing in 18 cases. Additionally, five cases were attributed to road traffic accidents, three to falls from height, one to blunt trauma, and one to another cause [25]. Bandyopadhyay observed that among the 49 patients' domestic falls in 27 cases, road traffic accidents in 10 cases, sports injuries in seven cases, and falls from bicycle in five cases [17]. Tandon et al. observed the various modes of injury like 47% at home [26]. The most frequent type of injury in the current study is road traffic accidents. The number of motor vehicles on the road is increasing, and traffic laws are not being properly enforced. Poor socioeconomic situation makes it difficult for working parents to watch over their kids, which has increased the number of traffic accidents.

In our study, the most frequently fractured bone was the femur, observed in nine cases (37.50%). This was followed by fractures of both bones in the forearm in seven cases (29.17%), the tibia in five cases (20.83%), the humerus in two cases (8.33%), and the ulna alone in one case (4.17%). A similar result was observed by many authors (fracture femur as the most common long bone involved). In their study of 30 cases of diaphyseal long bone fractures, Gavaskar and Singh observed 13 cases of femoral fractures (44%), nine cases of forearm fractures (30%), seven cases of tibia fractures (23%), and one case of humerus fracture (3%) [13]. Bandyopadhyay observed that among the 49 patients with long bone diaphyseal fractures, the femur accounted for 40, the tibia in six, and the humerus in three patients [17]. Khuntia et al. found that out of 30 pediatric long-bone fractures, there were five tibial fractures, eight forearm fractures, one humerus fracture, and 15 femur fractures [18]. In their investigation of 30 patients with diaphyseal fractures of long bones, Baig et al. found that 17 (56.7%) of the patients had femoral fractures, seven (23.3%) had tibial fractures, one (3.3%) had humeral fractures, and five (16.6%) had forearm fractures [11]. In their other investigations, Reddy and Dhaniwala found that long bones other than the femur were frequently affected [25]. In their investigation of long bone fractures, Furlan et al. noted 35 tibial, 42 forearm, 41 femoral, and 55 humeral fractures [10]. In their prospective investigation on the therapy of long bone fractures, Raut et al. noted that 50% of the forearm bone was affected, followed by the tibia (23.3%), femur (23.3%), and humerus (3.33%) [12].

In our study, the commonest location of fracture was the middle third (21 fractures, 67.74%), proximal third, and distal third accounted for five fractures each (16.13%). Various studies observed the middle third as the most common level of fracture. Gavaskar and Singh observed the level of fracture as middle third in 23 cases (77%), in the upper third in three cases (10%), and in the lower one-third shaft in four cases (13%) [13]. In their study, Raut et al. found that among the 30 patients, the majority had fractures of the middle one-third of the diaphysis (73.3%), while 26.7% had fractures of the upper one-third of the diaphysis [12]. During their study of diaphyseal fracture of long bones, Reddy and Dhaniwala observed the most common level of fracture as the middle third (59.52%) [25]. Flynn et al. noted that among the 58 fractures studied, the most common pattern observed was mid-shaft in 42 cases, with seven being distal and nine being proximal [8]. Bandyopadhyay observed fracture location in proximal one-third in five cases and middle one-third in 44 cases [17].

In our study, most of the fractures were seen on the right side (19 fractures, 61.29%), whereas 12 fractures (38.71%) were seen on the left side. A similar result of right-side involvement is noticed in many studies. Gavaskar and Singh reported right-side involvement in 18 patients (60%), while left-side involvement in 12 patients (40%) [13]. Raut et al. observed right-side predominance compared to the left-side (60% vs. 40%) [12]. Naorem and Temjensunep observed that 19 patients (63.33%) had right-sided fractures and 11 patients (36.67%) had left-sided fractures [14]. Bandyopadhyay in their study among 49 patients, the right side was involved in 27 cases (55.10%), and the left side was involved in 22 cases (44.89%) [17]. Ghilley et al. observed amongst the 14 cases, 10 cases (71%) were right-sided [21].

In our present study, transverse fracture accounted for a major portion of this series (18 fractures, 58.06%) followed by oblique fractures (eight fractures, 25.81%). Comminuted fractures accounted for five fractures (16.13%). Various studies observed similar results related to transverse fracture patterns. Naorem and Temjensunep reported amongst the 30 patients, 19 were transverse fractures (63.33 %), seven were oblique fractures (23.33%), four were segmental fractures (13.33%), and there were no comminuted fractures [14]. Reddy and Dhaniwala observed that transverse fractures accounted for the majority of instances (32 cases, 76.19%), with oblique fractures (six cases, 14.29%) coming in second [25]. In their investigation, Gavaskar and Singh found that 50% of the fractures were transverse, 20% were spiral, seven cases were short oblique, one case was segmental, and one case was comminuted [13]. Ghilley et al. observed that the majority of the patients in six cases (44%) had a transverse fracture pattern followed by a short oblique pattern in two cases (15%) and a long oblique in three cases (25%) [21]. Baig et al. discovered that 10 patients (33.33%) had transverse fractures, eight cases (26.7%) had comminuted fractures, seven cases (23.3%) had oblique fractures, and five cases (16.7%) had spiral fractures [11]. In their investigation, there were no segmental fractures. However, Kumawat et al. in their study of pediatric diaphyseal femur fractures among 30 patients observed an oblique pattern as the most common fracture type, occurring in 15 cases (50%), followed by transverse fractures in 12 cases (40%), with other patterns noted in three cases (10%) [27]. We found that transverse fractures were the most common and that TENS was the best way to stabilize them.

In our study, the majority of patients (20, 83.33%) underwent surgery within 10 days following the trauma, whereas four patients (16.67%) had an interval of more than 10 days. The observed average time between trauma and surgery was 6.41 days. Several studies examined the time between surgery and trauma. Baig et al. and Raut et al. investigated intramedullary elastic nailing for the treatment of long bone fractures, examining a total of 30 cases [11,12]. These findings revealed variations in the injury-to-surgery interval among the participants. Specifically, 10 patients (33.3%) underwent surgery within 24 hours of sustaining the injury. For a majority of cases, comprising 14 patients (46.7%), the injury-to-surgery interval ranged from one to two days. Additionally, a smaller subset of patients, totaling six (20%), experienced a longer delay, requiring more than two to three days before undergoing the surgical procedure. This insight into the timing of surgical interventions provides valuable information on the management and timelines associated with intramedullary elastic nailing in the context of long bone fractures. Bandyopadhyay found that among 49 patients with diaphyseal long bone fractures, the mean interval between injury and surgery was three days (range one to five days) [17]. Of the 31 fractures, open reduction had to be done in two fractures of both bone forearm (in the same patient), after unsuccessful attempts at closed reduction. Closed reduction was done in the majority of the fractures (29 fractures). Among the 537 patients with forearm fractures in the series, Fernandez et al. reduced 13 ulna and 18 radius fractures in an open manner [20]. Shams et al., in their use of TENS for treating femoral shaft fractures, discovered that while 44 fractures could be reduced closed, eight required open reduction [28]. In their analysis of 36 femoral shaft fractures, Rahman et al. found that while 31 fractures could be repaired with closed methods, five required open reduction [29].

In our study, out of 24 cases, the majority of patients (17 cases, 70.83%) had a hospital stay of more than seven days, while seven cases had a hospital stay of seven days or less. The mean hospital stay found in our study was 9.66 days (range six to 20 days). Our study result is comparable with Baig et al. who observed a mean duration of hospital stay of 11.6 days in their study of diaphyseal fractures across all bones among 30 patients [11]. In our study involving 24 cases, the majority of patients (16 cases, 66.67%) had a surgery duration between 46 and 60 minutes. Four patients (16.67%) underwent surgery lasting between 61 and 75 minutes, three patients (12.50%) had surgery completed within 30-45 minutes, and one patient (4.17%) had a surgery duration ranging from 76 to 100 minutes. The mean duration of surgery in our study was found to be 58.33 minutes. The mean operative time is comparable to that reported in studies by Raut et al. and Baig et al. [11,12]. In their conducted study, Raut et al. observed diverse durations for surgical times among the 30 patients undergoing a specific procedure. Specifically, five patients (16.7%) experienced surgical durations ranging from 30 to 45 minutes, while a larger proportion, comprising 12 patients (40%), fell within the 45- to 60-minute timeframe. Furthermore, seven patients (23.3%) underwent surgery with durations spanning 60 to 75 minutes, and the remaining six patients (20%) had operative times extending from 75 to 90 minutes. These findings provide a comprehensive overview of the variability in surgical durations among the participants, offering insights into the procedural aspects of the investigated medical intervention. The average operating time was 68 minutes [12]. Baig et al. noted a mean duration of surgery of 59.9 minutes in their study of diaphyseal fractures across all bones among 30 patients [11]. Bhat et al. and Lohiya et al. in their study of 73 cases of femoral shaft fractures noted average operative time as 37 (25-110) minutes [30,31]. Shams et al. studied 44 femoral shaft fractures and noted the mean time of surgery as 38 (30-45) minutes [28]. Kumar and Kisan in their study of fracture of femur noted the median duration of surgery as 45 minutes (30-75 minutes) [32].

In our study in 24 cases, the maximum number of patients (16 cases, 66.67%) had a duration of surgery between 46 and 60 minutes, four patients (16.67%) had surgery duration between 61 and 75 minutes, three patients (12.50%) surgery was completed in a range of 30-45 minutes while in one patient (4.17%) surgery duration ranged from 76 to 100 minutes. The mean duration of surgery in our study was found to be 58.33 minutes. The mean operative time is comparable to studies of Raut et al. and Baig et al. [11,12]. Within their investigation of 30 patients, Raut et al. found that seven (23.3%) and six (20%) had operative periods between 60-75 minutes and 75-90 minutes, respectively. Additionally, five (16.7%) and 12 (40%) patients had operative times between 30-45 minutes and 45-60 minutes, respectively [12]. A total of 58.83 minutes were spent on surgery. Baig et al. noted a mean surgery duration of 59.9 minutes in their study of diaphyseal fractures across all bones among 30 patients [11]. In their study of 30 patients with forearm fractures, Bhat et al. found that the duration of surgery varied from 25 to 45 minutes. Specifically, in cases requiring open reduction, the surgery lasted 45 minutes [30]. Lohiya et al. in their study among 73 cases of femoral shaft fractures noted average operative time as 37 (25 to 110) minutes [31]. Shams et al. [28] studied 44 femoral shaft fractures and noted the mean time of surgery as 38 (30-45) minutes. Kumar and Kisan [32] in their study of fracture of femur noted the median duration of surgery as 45 minutes (30-75 minutes).

Among the 31 fractures in our study treated with TENS, we observed complications in three fractures (9.68%). Two cases experienced entry site skin irritation (6.45%) accompanied by pain, while one case showed signs of delayed union (3.23%) with a 12-degree (varus) angular deformity and 2 cm of shortening. Amongst the two cases of entry site skin irritation, one case of fracture shaft femur right side in a 15-year-old female with a fracture in the middle one-third shaft presented with irritation on the lateral aspect of distal femur right side (at entry site) with restriction of knee range of motion during follow-up. The fracture was united by 24 weeks. Entry-site skin irritation was relieved with analgesics and implant removal. The patient was advised to do regular physiotherapy. Later, it improved with satisfactory results at the end of 39 weeks of follow-up. In another case, a 13-year-old male patient with a fracture proximal one-third of the shaft femur right side presented with irritation on the lateral aspect of the distal femur right side (at entry site) with restriction of knee range of motion during follow-up. The fracture union was seen for 24 weeks. He was treated with analgesics and physiotherapy with satisfactory results for 39 weeks of final follow-up. The patient was advised of implant removal. Shams et al. [28] in their study of 52 pediatric femoral shaft fractures managed by TENS various complications were noted in 22 cases. They observed entry site skin irritation/bursitis in eight cases due to friction caused by cut ends of the nail. The nails were removed after an average of 47 weeks (40-54 weeks range). Flynn et al. examined 58 cases of pediatric femur fractures and observed soft tissue irritation in five cases. Consequently, in one instance, the nail had to be removed earlier, within a period of four to 10 weeks [8].

In one case we observed delayed union, the patient was a 15-year-old male with an oblique fracture of the distal third shaft femur of the right side operated with two TENS of 3.5 mm each. Postoperatively and during follow-up X-rays, minimal distraction was observed at the fracture site. There was no infection at the fracture site/no skin irritation or bursitis. On subsequent follow-up, the fracture was united in minimal varus with angulation of 12 degrees and 2 cm of shortening. The resulting outcome was poor at the end of the final follow-up. Bandyopadhyay in his study of 49 patients observed delayed union in two patients which took 24 weeks for union [17]. Moroz et al. assessed a total of 229 children with 234 fractures of the diaphysis of the femur and observed delayed union in two cases (0.9%) [33].

In our investigation, the duration of follow-up varied from six to 39 weeks. Three patients finished the 12-week follow-up, eight patients finished the 24-week follow-up, and 12 patients finished the 39-week nine-month follow-up. For six weeks, one patient was monitored. The average follow-up period was 29.25 ± 11.02 weeks. Various studies have reported differing average follow-up periods. Reddy and Dhaniwala found an average of 22.08 weeks [25]. Khuntia et al. recorded a longer average follow-up duration of 28 months [18]. Bandyopadhyay reported an average follow-up period of 18.8 months [17]. Meanwhile, Furlan et al. observed the longest average follow-up period among the studies, at 41.3 months [10].

We have used the scoring criteria described by Flynn et al. for evaluating the results of our study [8]. Results were evaluated at the end of the last follow-up. A total of 31 fractures in 24 children were included for outcome evaluation at the final follow-up. Only one fracture (3.23%) had poor outcome. 

Limitations

Our study has limitations, as the sample size and follow-up period were limited. The outcome of our study compared with various other studies is depicted below in Table 2.

**Table 2:** Outcome in various studies compared to the present study

Studies	Results		
	Excellent	Satisfactory	Poor
Present study	90.32%	6.45%	3.23%
Flynn et al. [8]	65.5%	31.03%	1.72%
Raut et al. [12]	83.3%	16.7%	-
Bandyopadhyay [17]	82%	18%	-
Reddy and Dhaniwala [25]	76.92%	17.95%	0.13%
Baig et al. [11]	73.33%	26.67%	-
Vaish et al. [34]	73%	27%	-

## Conclusions

For patients aged five to 16, this is a safe, straightforward, uncomplicated, quick, dependable, and efficient way to treat long bone fractures in children. It requires less time during surgery and a suitable amount of time for the bone to recover. It notably shortens hospital stays and does away with the requirement for extended bed rest. It provides elastic mobility, stability, and fast union at the fracture site, all of which are perfect for early mobilization. It is linked to a low incidence of complications and a high degree of functional results.
